# Identification of trichlormethiazide as a Mdr1a/b gene expression enhancer via a dual secretion-based promoter assay

**DOI:** 10.1002/prp2.109

**Published:** 2015-01-05

**Authors:** Sarina Schulze, Sven Reinhardt, Christian Freese, Ulrich Schmitt, Kristina Endres

**Affiliations:** 1 Clinic of Psychiatry and Psychotherapy, University Medical Center Johannes Gutenberg UniversityMainz, Germany; 2 REPAIR-lab, Institute of Pathology, University Medical Center of the Johannes Gutenberg University Mainz and European Institute of Excellence on Tissue Engineering and Regenerative MedicineMainz, Germany

**Keywords:** Blood–brain barrier, endothelial cells, P-glycoprotein, promoter

## Abstract

Transporters of the ATP-binding cassette (ABC) family such as MDR1 play a pivotal role in persistence of brain homeostasis by contributing to the strict permeability properties of the blood–brain barrier. This barrier on one hand compromises treatment of central nervous system diseases by restricting access of drugs; on the other hand, an impaired or altered function of barrier building cells has been described in neurological disorders. The latter might contribute to increased vulnerability of the brain under pathological conditions or even enforce pathogenesis. Here, we present a novel approach for a systematic examination of drug impact on Mdr1 gene expression by establishing a dual reporter gene assay for the murine upstream core promoters of Mdr1a and b. We validated the time-resolved assay in comparison with single reporter gene constructs and applied it to analyze effects of a Food and Drug Administration (FDA)-approved drug library consisting of 627 substances. The chemo-preventive synthetic dithiolethione oltipraz was reidentified with our assay as an already known inducer of Mdr1 gene expression. Together with two newly characterized modifiers – gemcitabine and trichlormethiazide – we prove our findings in a blood–brain barrier culture model as well as in wild-type and Mdr1 knockout mice. In sum, we could demonstrate that our dual reporter gene assay delivers results, which also persist in the living animal and consequently is applicable for further analysis and prediction of Mdr1 regulation in vivo.

## Introduction

Blood–tissue barriers exclude macromolecules from the systemic blood flow to enter or to persist in the respective tissue. Examples for such barriers are the blood–brain, the blood–spinal cord, and the blood–testis barrier (Bartanusz et al. 2011; Franca et al. 2012; Obermeier et al. 2013). The blood–brain barrier (BBB) tightly regulates passage of molecules from the blood into the brain and vice versa. It is formed by three main cell types that are in close contact and are embedded in a basal lamina: endothelial cells of the blood vessel walls lined by pericytes and astrocytes from the brain parenchyma. Physiological function of this so-called neurovascular unit guarantees homeostasis of the brain by building up a paracellular and transcellular barrier. Endothelial cells, for example, express efflux transporters that eliminate potential harmful substances from the central nervous tissue (Giacomini et al. 2010). These transporters belong to the family of ATP-independent SLC (solute carriers, reviewed in (He et al. 2009)) or to the ATP-utilizing transmembrane proteins of the ATP-binding cassette (ABC) class (ABC transporters, e.g., [Vasiliou et al. 2009]). The latter comprises 48 functional transporters in humans with P-glycoprotein (PGP/MDR1/ABCB1) as the first to be cloned and analyzed in detail (Ueda et al. 1987a,b). While human PGP is encoded by a single gene on chromosome 7q21 (Callen et al. 1987), rodents display a gene duplication (chromosome 5, (Gros et al. 1988; Raymond et al. 1990) that gives rise to two distinct mRNAs designated as Mdr1a and Mdr1b. Specialization of the two rodent proteins is assumed to result in shared but also in isoform-specific substrate spectra that together reflect the phenotype of the human homologue (Devault and Gros 1990; Silverton et al. 2011).

Increased expression of drug efflux transporters at the BBB has been proposed as a mechanism responsible for multidrug resistance, hampering curative approaches in neurological disorders such as glioblastoma multiforme (Demeule et al. 2001), (Loscher and Potschka 2005). For example, an increased expression of PGP mRNA and protein in brain tissue from patients with Temporal lobe epilepsy was described (reviewed in Aronica et al. 2012). Interestingly, PGP overexpression was already observed in malformations of cortical development before the onset of seizures (Sisodiya et al. 1999) and only within the lesions and not in peri-lesional tissue (Aronica et al. 2003) indicating a direct contribution to pathogenesis. For Alzheimer’s disease such a direct pathomechanism has been postulated: pharmacological blockade of PGP resulted in a decrease of extracellular levels of Abeta peptides (Lam et al. 2001) and altered frequencies for SNPs within the respective gene have been found in a subpopulation of demented patients as compared to healthy controls (Cascorbi et al. 2013). Therefore, elucidating regulatory mechanisms that control gene expression, translation and function of PGP have to be taken into account in therapeutical or preventive strategies for neurological diseases.

Reports exist on regulation of MDR1 gene expression via transcription factors such as SMRT (silencing mediator for retinoid and thyroid receptors Hirooka-Masui et al. 2013), Egr-1 (Tao et al. 2013), or E2F1 and EAPP (E2F-associated phospho-protein Andorfer and Rotheneder 2013). Additionally, activation of xenosensors such as CAR or PXR (constitutive androstane receptor, pregnane x receptor) has been demonstrated to dynamically alter MDR1 transcription in drug processing organs (reviewed, e.g., in Wang and LeCluyse 2003). Treatment of HepG2 cells with valproic acid, for example, has been shown to increase MDR1 mRNA amount in the presence of overexpressed CAR or PXR (Cerveny et al. 2007). This was accompanied by enhanced occupancy of the respective responsive elements on MDR1 promoter by CAR/RXR heterodimers. Besides ligands for nuclear receptors, single drugs that influence MDR1 transcription have been identified with yet unknown mechanistic properties. For example, mollugin (from *Rubica cordifolia* L.) inhibits MDR1 expression in MCF-7 cells (Tran et al. 2013). This has also been demonstrated for the approved drug temozolomide used in therapy of Glioblastoma multiforme (Riganti et al. 2014). To our knowledge, a systematic, experimental approach for identifying MDR1-modifying drugs is still missing. For this purpose, we established a high-throughput-adoptable systematic screening of drugs influencing murine Mdr1 gene expression. Therefore, we used the core downstream promoter regions of the respective *M. musculus* genes (Hsu et al. 1990; Cohen et al. 1991) in reporter gene constructs encoding secreted luciferases and evaluated selected candidates from an Food and Drug Administration (FDA)-library screening in vitro and in vivo.

## Materials and Methods

The FDA-approved drug library was from Enzo Lifesciences (Farmingdale, NY); selected drug candidates, oltipraz, trichlormethiazide, gemcitabine as well as trichostatin A (TSA), were from Sigma Aldrich. All substances were dissolved in dimethylsulfoxide (DMSO) with a maximum volume ratio of 0.1% in cell culture medium. Human p53 and HSF1 expression vectors were obtained from Origene (Rockville, MA).

### Cloning of Mdr1a and b promoter reporter plasmids

Primers for amplification of the promoter regions via PCR from chromosomal murine DNA (FVB/N strain) were as follows: Mdr1a_Pro_for: 5′-CAATTGGACTCTGCAAGTGTGTCTC-3′/Mdr1a_Pro_rev: 5′-GGATCCACCTCACGTGCCACCTCCG-3′; Mdr1b_Pro_for: 5′-CAATTGGCTATGTCAGGGAAAGTGTC-3′/Mdr1b_Pro_rev: 5′- GGATCCACCTCACGTGCCACCTC-3′. Both sequences were first inserted into pUC19 via TA cloning. The CMV promoter of the vector pCMVGLuc (NEB, Ipswich, MA) was replaced by the murine Mdr1a or Mdr1b promoter sequence (−252 bp to -137 bp; −296 bp to -144 bp) cut from respective pUC19 constructs using *Mfe*I and *BamH*I restriction sites generated by PCR primers (underlined in the primer sequences). In case of the Mdr1a promoter reporter plasmid, the *Gaussia* luciferase cDNA was subsequently replaced by the cDNA sequence of the *Cypridina* luciferase derived from the pClucBasic2-vector (NEB) by *BamH*I and *Not*I digestion to obtain pMdr1a-CLuc. A mock vector (pGLuc) was constructed by removing the CMV promoter region from the pCMVGLuc-vector (NEB) through digestion with *Mfe*I and *BamH*I digestion, followed by T4 DNA polymerase incubation to obtain blunt ends for re-ligation. All sequences were verified by restriction analysis and sequencing (SRD, Bad Homburg, Germany).

### Cell culture and transfection

N2A cells (ATCC: CRL 131) were chosen as a murine neuronal-like cell model because they are known to have a relatively stable phenotype and furthermore overexpress Mdr1 (e.g., Nicolae et al. 2013). Cells were grown in DMEM (Life Technologies, Darmstadt, Germany) supplemented with 10% heat-inactivated fetal bovine serum and 1% glutamine (both GE Healthcare, Piscataway, NJ) in a humidified atmosphere of 95% and 5% CO_2_ at 37°C. The cells were cultured in 10-cm dishes. When confluence was achieved, cells were passaged at a rate of 1:10.

Transient retrotransfection using Lipofectamine2000 (Life Technologies) was performed as recommended by the manufacturer. In brief, 200 ng of DNA and 0.3 *μ*L Lipofectamine were applied in OptiMEM (Life Technologies) per well of poly-l-Lysin-coated 96-well plates. Cells were seeded at a density of 30,000 cells per well on the lipofection mixture (white 96-well plates with glass bottom, Greiner [Frickenhausen, Germany], precoated with poly-l-lysin, Sigma [St. Louis, MA]) and incubated for 7 h. In case of the screening approach, cells were treated with one of the 627 substances derived from a FDA-approved drug library (Enzo LifeSciences) or DMSO as solvent control (0.1% v/v) upon finished transfection period. Drugs are delivered as stock solutions of 2 mg/mL, final dilutions were chosen in dependency on a toxicity assay (data not shown) with an initial dilution of 1/3000. If drugs displayed toxic effects, they were further diluted giving final ratios of 1:75,000, 1: 150,000, or 1:300,000. For 13 drugs of the compound library consisting of 640 drugs, toxic effects were observed even at this high dilution ratios, and therefore, they were excluded from testing. TSA was added in a final concentration of 15 nmol/L as a positive control. After 24 and 48 h of incubation, the luminescence of both enzymes was measured in the collected supernatants by a dual luciferase assay (see below).

### Promoter assays

For all reporter gene assays, we used the enzymatic reaction of two individual secreted luciferases: *Gaussia* luciferase from the marine copepod *Gaussia princeps* and *Cypridina* luciferase from the marine ostracod *Cypridina noctiluca*. Both enzymes are secreted due to a naturally occurring signal peptide (Barnes and Case 1972; Nakajima et al. 2004) and are therefore appropriate for repeated measurements from cell supernatant. Luciferase catalytic activity measured by light emission (Fluostar Optima; BMG) correlates proportionally to transcriptional activity of the promoters of Mdr1a and Mdr1b, respectively.

In case of separate measurements of either *Gaussia p*. or *Cypridina n*. luciferase, 10 *μ*L of cell culture supernatant were mixed with the respective substrate by automated injection using the Fluostar Optima (BMG). We assessed an optimal final substrate concentration for the *Gaussia p*.-derived enzyme of 20 *μ*mol/L in PBS (Roth, Karlsruhe, Germany) and 3 *μ*mol/L of *Cypridina n*. luciferase substrate in PBS (NanoLight, Pinetop, AZ). Both substrates were stored as stock solutions at −80°C, the *Cypridina* luciferase was kept under argon atmosphere and sodium ascorbate was added at 0.3 mol/L as an oxidation protection during storage.

In addition, we established a dual reporter assay for consecutive measurement of the murine Mdr class1 promoter activities. Principle of the assay is the initial application of *Gaussia* substrate, measurement for 10 sec (activity of Mdr1b promoter) and a subsequent quenching of the photon emission derived from this first enzymatic reaction. This is accomplished by injecting the *Cypridina* luciferase substrate supplemented with SDS. Activity of the Mdr1a promoter is then measured for another 10 sec (Fig.2B). Wu et al. (2007) formerly reported that *Gaussia* luciferase activity is inhibited by application of SDS in a concentration of 0.1%. We tested a series of SDS concentrations and were able to gain a 100% loss of *Gaussia* luciferase-dependent photon emission with 0.01% SDS, while *Cypridina* luciferase was only slightly affected (about 3% signal reduction, see Fig.2).

All luminescence measurements were normalized to protein content of the respective cell lysate (quantitation performed using Rot-Nanoquant, Roth and Biochrom Asys Expert 96-Microplate Reader) despite in case of the dual screening approach. For this, representative plates were analyzed for protein concentrations in cell lysates to demonstrate lack of proliferative or toxic influence of the compounds from the FDA-approved drug library (data not shown).

### Preparation of whole-cell lysates and immunoblotting

N2A cells were transiently transfected with the respective expression plasmid (pCMVp53, pCMVHSF1 or empty vector [mock]) or treated with TSA as described above. Cells were lysed in passive lysis buffer and samples substituted with LDS sample buffer (Life Technologies) containing 10% dithiothreitol (1 mol/L, Roth, Karlsruhe, Germany). Samples were incubated for 10 min at 95°C and stored at −20°C. Protein of whole-cell lysate derived from one well each were separated on 8 or 14% SDS-polyacrylamide gels (p53/HSF1 or Histones) and blotted onto nitrocellulose membrane at 100 V for 2 h. Immunodetection was carried out by blocking the membranes for 1 h in blocking solution and incubation overnight at 4°C with the appropriate primary antibody at a dilution of 1:1000. Antibodies were as follows: anti-p53 (ab16465; Abcam, Cambridge, UK), anti-HSF1 (ab52757; Abcam), and anti-pan-H3 or anti-AcH3 (NEB). Blots were incubated with respective secondary antibody coupled with horseradish peroxidase (Thermo Scientific, Karlsruhe, Germany) and signals obtained by applying SuperSignal West Femto chemiluminescent substrate (Thermo Scientific) were captured using a CCD camera imaging system (Raytest, Straubenhardt, Germany).

### PGP-ATPase assay

The PGP-ATP Assay (SOLVO Biotechnology USA Inc. [Boston, MA]) was used to analyze potential substrate characteristics of the three evaluated PGP expression modulators (oltipraz, trichlormethiazide, and gemcitabine). Within the assay, which was performed as suggested by the manufacturer, the drugs were applied in concentrations as indicated. DMSO served as a solvent control and was used at a dilution of 2%. Verapamil served as a positive control, representing a strong PGP substrate (e.g., Spoelstra et al. 1994). All values were calculated as % of those obtained for verapamil.

### Transport assay

Primary porcine brain endothelial cells (PBEC) were prepared as described before (Freese et al. 2014) and seeded directly after preparation on transwell filter plates (PET, 0.4 *μ*m pores; Corning). Eight days after seeding, cells were treated with oltipraz, trichlormethiazide, or gemcitabine or the solvent DMSO for 48 h (final concentrations: 5; 2.5; 0.35 *μ*mol/L). Optimal concentrations were determined by cellular toxicity assays before (MTT, LDH release, and ATP content quantitation, data not shown). Integrity of the cell barrier at start point of the experiment was verified by TEER measurement using chop stick electrodes: TEER values >100 Ohm × cm^2^ were accepted for performing the transport experiment. After 48-h incubation with the potential PGP expression modulators, 50 *μ*L of the cell supernatant of the upper compartment of the transwell filter plate was replaced by 50 *μ*L risperidone containing medium (Risperdal®, 0.2 mg/mL; Janssen-Cilag [Neuss, Germany]). Three hours later, 150 *μ*L cell supernatant from the upper compartment and 300 *μ*L from the lower compartment were collected and stored at −20°C until measurement by HPLC.

### Animal treatment

Male FVB/N and Mdr1a/b knockout mice (Schinkel et al., 1997) from the animal facility of the University Medical Center of Mainz (25–45 g) were used. Animals were housed in groups of 2–5 with free access to food and water. A 12-h light–dark cycle (6 am to 6 pm light on) was maintained at a temperature of 22°C and a relative humidity of 60%. All experiments were conducted in accordance with the official regulations for the care and use of laboratory animals and approved by local authorities. Substances were injected intraperitonally on two consecutive days as follows: oltipraz 30 mg/kg body weight, trichlormethiazide at 8 mg/kg, and gemcitabine at 20 mg/kg. All substances were solved in DMSO and were applied at max. 1.6 mL/kg body weight. DMSO-treated mice served as a control. On the third day, 3 mg/kg risperidone (Janssen-Cilag) was injected i.p. and mice were sacrificed 3 h later. Brains were dissected, the cerebellum discarded and one hemisphere snap frozen, and stored subsequently at −80°C until used for HPLC analysis. The other hemisphere was immediately placed in 1 mL of RNAlater (Qiagen, Hilden, Germany) for RNA preparation. Truncal blood was drained from mice and serum collected by centrifugation. Serum was stored at −80°C until further analysis.

### HPLC analysis of cell culture supernatants, serum, and brain tissue samples

Risperidone and its metabolite 9-OH-risperidone were quantified by HPLC as described before (Kirschbaum et al. 2008). Cell supernatants were adjusted to a total volume of 300 *μ*L per sample by addition of cell culture medium. Serum of experimental animals was supplemented with human risperidone-free plasma to adjust volume to 300 *μ*L if needed. Brain hemispheres were extracted in methanol (volume [mL] = 4 × brain weight [mg]) using a tissue mill and stainless steel beads (Qiagen), centrifuged and supernatant collected for HPLC analysis (preparation described in detail in [Doran et al. 2005; Kirschbaum et al. 2008]).

### RNA preparation and Real-Time RT-PCR

RNA was prepared from tissue stored in RNAlater at −80°C following the protocol of the manufacturer (lipid tissue midi kit; Qiagen). Concentration and purity of samples were assessed using a NanoDrop spectrophotometer (Pierce, Wilmington, DE). RNA was diluted to 50 ng/*μ*L and applied to Real-Time RT-PCR using the QuantiTect SYBR Green One Step RT-PCR Kit (Qiagen) and the following primers: abcb1a QT01753416, abcb1b QT00140945, gapdh QT00309099 (all Qiagen). RT-PCRs were performed on a StepOnePlus Cycler (Applied Biosystems, Darmstadt, Germany) with 100 ng RNA per reaction. Relative quantities were calculated using appropriate standard curves.

### Statistical analysis and bioinformatics

Testing of statistical significance was performed using one-way ANOVA followed by Bonferroni posttest or by unpaired Student’s *t*-test. Values of *P* < 0.05 were considered statistically significant. Analysis of transcription factor (TF) binding sites was performed using MatInspector (Genomatix) with a minimum matrix and core similarity of 0.75.

## Results

### Characterization of Mdr1a and b promoter vectors

**Figure 1:**
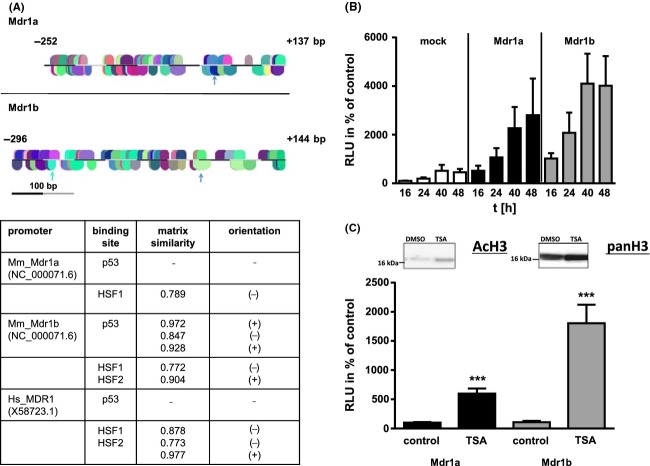
Characterization of Mdr1a and 1b reporter constructs. (A) Core promoter regions of Mdr1a and b according to literature (Hsu et al. 1990; Cohen et al. 1991). An in silico analysis revealed about 60–70 transcription factor binding sites in both sequences (MatInspector Analysis). Predicted binding sites for p53 (green) and HSF1 (blue) are indicated by arrows and given in detail in the table below. For comparison, we included HSF-binding sites of the human promoter region. As HSF1 and 2 are able to form heterocomplexes (Lecomte et al. 2013), we also included information for this transcription factor. (B) Basal activity of both promoters was measured using single *Gaussia*-based reporter plasmids. N2A cells were transfected and cell supernatant with secreted enzyme collected at the indicated time points. RLU (relative light units) are given as mean ± standard deviation from three independent experiments performed in triplicate. Cells transfected with the promoter-less control vector (mock) were used as a control and values obtained for those cells at 16 h post transfection were set to 100%. All data obtained for promoter-containing constructs were significantly higher than the respective control sample (one-way ANOVA, Bonferroni posttest, *P* < 0.05). (C) Induction of either promoter was sustained by applying 15 nmol/L TSA to cells after finishing transfection period for another 16 h. Values represent mean ± standard deviation from three independently conducted experiments (*n* = 9, Student’s *t*-test; ***P < 0.001). The potential of TSA to induce histone acetylation is demonstrated by western blotting of corresponding cell lysates (usage of pan-H3- or AcH3-specific antibody).

Hsu et al. (1990) reported that within brain, heart, and lung Mdr1a promoter activity is not observed but other publications described an influence of Mdr1a on blood–brain barrier properties (Desrayaud et al. 1998). Due to this discrepancy we chose for our investigation the core region of both, murine Mdr1a and b downstream promoters as described by Hsu et al. (1990) and by Cohen et al. (1991). Using MatInspector analysis (Genomatix), we identified 71 potential TF binding sites in the Mdr1a promoter and 67 in the Mdr1b promoter region (Fig.1A). Interestingly, comparison of the promoter regions with the respective human sequence resulted in shared transcription factor binding sites such as CAAT boxes or GC boxes (not shown) but also in individual binding sites. For example, all three promoters included binding sites for the two major heat-shock factors HSF1 and/or 2 but only Mdr1b contained a predicted p53 binding site (Fig.1A).

Time-resolved measurement of basal promoter activities in N2A cells revealed that the Mdr1a promoter-driven reporter evoked comparably weaker signals than the Mdr1b reporter (both reporter constructs encoding *Gaussia* luciferase Fig.1B): 48 h after transfection the Mdr1a reporter resulted in a basal activity of 2400% as compared to empty vector transfected cells, while Mdr1b reporter yielded a relative activity of 3600%. Comparable results were obtained in a second cell line (SH-SY5Y, data not shown). This is in accordance with reports from literature that demonstrated murine Mdr1a being less active than Mdr1b in the absence of exogenous enhancers (Hsu et al. 1990; Cohen et al. 1991). To test the potential induction of both promoters, we applied TSA, a histone deacetylase inhibitor, to cells transiently transfected with either reporter construct. Upon 24 h of treatment, we observed strong induction of both reporters: Mdr1a-vector-transfected cells gained a signal of 500% compared to solvent-treated cells and Mdr1b-vector-transfected cells resulted in an increase to 1700% (Fig.1C). This resembles previous reports which in sum conclude epigenetic regulation of PGP gene expression by histone acetylation status (e.g., Jin and Scotto 1998; Balaguer et al. 2012; Henrique et al. 2013).

### Identification of Mdr1a and b transcriptional inducers by a dual promoter assay approach

**Figure 2:**
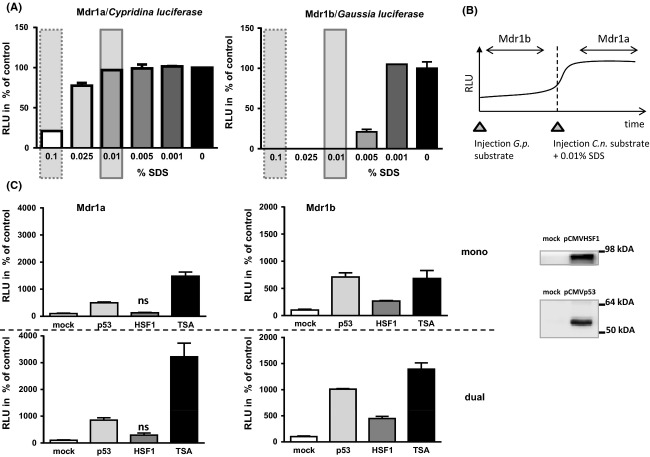
Definition of experimental parameters and evaluation of the Mdr1 dual promoter assay. (A) Measurement of luciferase enzymatic activity in dependency of SDS supplementation. Supernatants from cells transfected with the respective reporter were supplemented with SDS as indicated (v/v). Water served as a solvent control (0% SDS) and values were normalized to this control (mean ± standard deviation from technical replicates). (B) Experimental setup for dual reporter gene assay. Cell supernatants were first supplemented with *Gaussia p*. substrate and light emission detected for 10 sec. The signal was subsequently quenched by addition of 0.01% SDS in *Cypridina n*. substrate mixture, followed by another 10-sec detection period. (C) Comparison of single- and dual reporter gene assay. To demonstrate reliability of the newly established dual reporter gene assay for Mdr1a and 1b, cells were cotransfected with both reporter gene constructs and transcription factor expression plasmids or empty vector (mock). Overexpression of transcription factors was proven by western blotting of respective cell lysates (see right part of the figure). Additionally, cells only transfected with reporter constructs were treated with TSA (15 nmol/L). Supernatants were collected and either luciferase activities were measured separately (mono) or in the dual setup. Values are given as mean ± standard deviation from three independently conducted experiments (*n* = 9, one-way ANOVA, Dunnett’s multiple comparison test; all data have a *P* < 0.05 as compared to mock if not indicated otherwise).

Both *Gaussia* luciferase reporter vectors – Mdr1a and b promoter-driven – yielded reliable activity as compared to mock vector and in relation to each other as well as upon induction (Fig.1B and C). Therefore, we established a dual promoter assay based on these two core promot-ers regulating *Gaussia* luciferase (Mdr1b promoter) and *Cypridina* luciferase (Mdr1a promoter). It has already been described that *Gaussia* luciferase is quenchable by SDS administration (Wu et al. 2007). Different concentrations of SDS were added to supernatants obtained from cells transfected with Mdr1a-*Cypridina* luciferase or Mdr1b-*Gaussia* luciferase and residual activity of either enzyme was measured. 0.1% SDS was able to fully suppress *Gaussia* luciferase enzymatic activity as reported by Wu et al. (2007). Nevertheless, this concentration also reduces *Cypridina* luciferase activity to about 20% of control-treated supernatants and therefore was not applicable for the dual assay (Fig.2A). Administration of SDS in a final concentration of 0.01% was sufficient to completely quench *Gaussia* luciferase activity and resulted only in a 3% reduction of *Cypridina* luciferase-evoked signal. In consequence, we chose a final concentration of 0.01% SDS for our assay with sequential injection of both luciferase substrates (Fig.2B).

To test reliability of the dual reporter assay, we cotransfected the reporter plasmids with potential or known inducers of either promoter activity (p53 and HSF1, overexpression confirmed by western blotting, see Fig.2C) or treated cells with TSA. Luminescence signals were obtained from singular (“mono,” Fig.2C) as well as from measurements with subsequent addition of both enzyme substrates (“dual”). In sum, all results obtained with singular measurements were identical to those observed in the dual assay, but effect sizes were even higher in the dual assay. For example, p53 increased Mdr1a promoter activity in the singular assay to 400% of control, while 750% were measured in the dual assay. HSF1 only induced Mdr1b promoter (singular assay: 170% of control; dual: 350% of control), while Mdr1a promoter activity remained unaffected.

**Figure 3:**
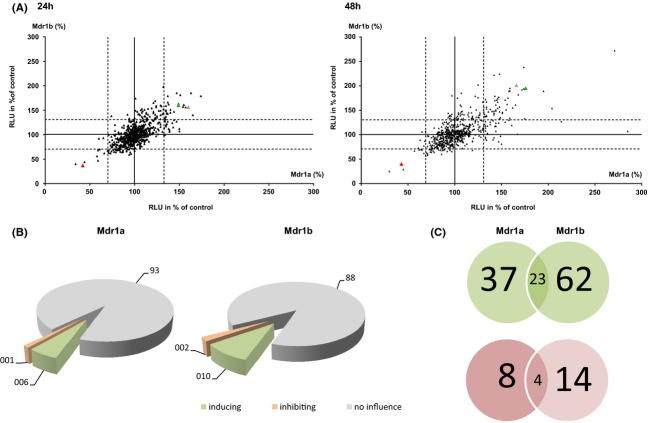
Screening of FDA-approved drugs for Mdr1a and b expression modifying potential. (A) Mdr1a and b promoter activities upon 24 or 48 h of incubation with respective substances. N2A cells were transiently cotransfected with Mdr1a- and Mdr1b promoter reporter and after end of transfection period (7 h) drugs of the FDA-approved compound library were added in fresh culture medium. After 24 or 48 h of treatment, cell supernatant aliquots were collected and subjected to dual luciferase reporter gene assay. RLU were normalized to values of DMSO-treated control cells. TSA (15 nmol/L) served as an internal positive control (data not shown). All substances were tested in three independent experiments; drugs that resulted in experimental data with a standard deviation of >30% were retested in two additional experiments. Values are given as means, standard deviations are not included in the graph for reasons of clarity (red: gemcitabine; green: trichlormethiazide; gray: oltipraz). (B) Outcome of screening. Drugs were defined as hits (inhibitors or activators of the respective promoter activity) when RLU were obtained >130% or <70% of solvent control for both time points, 24 and 48 h. C: Modifier spectra of Mdr1a and b. Selected hits were analyzed for their potential to induce/inhibit a single or both promoter activities.

Our newly established dual assay for Mdr1a and b promoter activity revealed reproducible and reliable data as compared to single measurements and observations gained from literature. Therefore, we next screened a library of FDA-approved drugs for potential modulators of Mdr1 expression by applying the dual promoter assay in N2A cells: 627 substances were tested for their ability to influence Mdr1a or b-driven reporter activity upon 24 or 48 h of treatment (for applied concentration see Table S1). Hits were defined by at least 30% increase or decrease of promoter activity (Fig.3A). Ninety-three percent of all tested drugs revealed no influence on Mdr1a promoter activity, while 88% had a neutral effect on Mdr1b (quantitation of substances with a comparable effect at 24 or 48 h of observation; Fig.3B). 5.9% of all substances were able to enhance Mdr1a promoter activity, while 1.3% decreased it. For Mdr1b, 9.8% elevating drugs were obtained and 2.2% with an attenuating effect. Interestingly, only 23 of all activating drugs and four of all inhibiting ones were common for both reporter measurements (Fig.3C, for a complete list, see Table S1). Interestingly, by assessing EC/IC_50_ values for treatment with three exemplary substances, oltipraz, trichlormethiazide, and gemcitabine, both promoters responded with different sensitivities (see Fig. S1).

### Evaluation of selected candidates from the screening in vitro and in vivo

**Figure 4:**
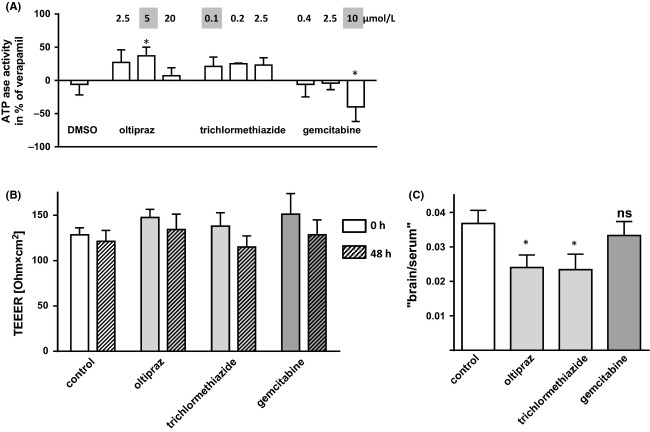
Transport characteristics of selected hits. A: PGP substrate properties of drug candidates. Selected drugs from the screening approach were subjected to an in vitro PGP activity assay using membranes from heterologous Mdr1b expressing insect cells. Results from at least duplicates were normalized to verapamil-treated samples (gray boxes indicate reported maximal human plasma concentrations; (Sketris et al. 1981; Benson 1993; Shord et al. 2003). Statistical analysis was performed using one-way ANOVA, Dunnett’s multiple comparison test (*P < 0.05 as compared to DMSO). (B) Integrity of endothelial cell barrier under compound treatment. Primary porcine cells (PBEC) were seeded and TEER was measured as a sign of barrier integrity with chop stick electrodes before (bars without pattern) and after 48 h of incubation (striped pattern). Statistical analysis (one-way ANOVA, Dunnett’s multiple comparison test) revealed no differences between DMSO and single treatments as well as begin of treatment to end of incubation period. (C) Influence of selected drugs on risperidone transport via PBEC derived in vitro barrier. PBEC were seeded on a transwell system and treated for 48 h with the respective drugs. Risperidone was added for 3 h. Subsequently cell supernatant samples were conserved from the upper compartment (“serum”) and from the lower compartment (“brain”). Risperidone concentrations were determined by HPLC and ratios calculated for transport efficacy (unpaired Student’s *t*-test versus control; *P < 0.05, ns, *P* > 0.05). 9-OH-risperidone was not detectable by HPLC in this cell culture model.

To substantiate the results from our cell culture model-based screening approach, we selected one known inducer of either promoter activity and two newly identified modifiers: oltipraz has been shown to enhance Mdr1a as well as Mdr1b gene expression in rats upon 4 days of treatment with 150 mg/kg (Merrell et al. 2008) and led to a 1.7- and fourfold induction of mRNA levels. In our screening approach using murine Mdr1 promoter sequences and N2A cells, we obtained similar values (a 1.7- and a twofold increase in promoter activity, Fig.3A, not highlighted) and therefore chose oltipraz as a control substance for further in vivo investigations. Trichlormethiazide evoked a 1.6 and a 1.8 increase (mean of promoter activities at 24 and 48 h) for Mdr1a and 1b and therefore represented a new general gene expression enhancer. Gemcitabine on the contrary decreased both promoter activities to about 40% of solvent-treated cells. First, we tested all three substances regarding their properties as PGP substrates in an in vitro test system to reveal that no major interference with transport despite gene expression will occur. For gemcitabine, it has been already reported that it does not act as a PGP substrate (Ogawa et al. 2005). In accordance with these data, we obtained measurement values comparable to the solvent control (Fig.4A) and only the highest concentration exhibited an inhibitory potential of gemcitabine in the respective assay system. Oltipraz as well as trichlormethiazide displayed weak substrate activity as compared to the positive control verapamil but this did not reach significance for trichlormethiazide at all tested concentrations. For oltipraz, 5 *μ*mol/L indicated a substrate activity, while for lower concentrations (2.5 *μ*mol/L) ATPase activity did not reach significance (*P* = 0.06) and a concentration of 20 *μ*mol/L had no enhancing impact on ATPase activity.

To evaluate the potential of the selected drug candidates to interfere with transport capability, we used an in vitro model of the blood–brain barrier with primary porcine endothelial brain cells (PBEC, as e.g., described in [Freese et al. 2014]). Substances were applied to PBECs seeded on a filter well system for 48 h and risperidone was added for the last 3 h as a well-characterized substrate of PGP (Kirschbaum et al. 2008). If a drug would interfere with the amount/activity of transmembrane proteins involved in risperidone transport, this should, for example, result in decreased entry into the “brain” compartment for a Mdr1/PGP expression enhancer. TEER value measurement before application of substances and at the end of the test period confirmed that barrier properties were not affected by the respective drug (Fig.4B). When compared with solvent control, oltipraz as well as trichlormethiazide decreased the transport of risperidone to the lower compartment of the transwell system significantly by about 30% (Fig.4C) which indicates an altered transport capacity of PGP molecules expressed in the endothelial cells. Gemcitabine on the contrary showed no influence on the transport rate in this cell culture model (*P* = 0.58).

**Figure 5:**
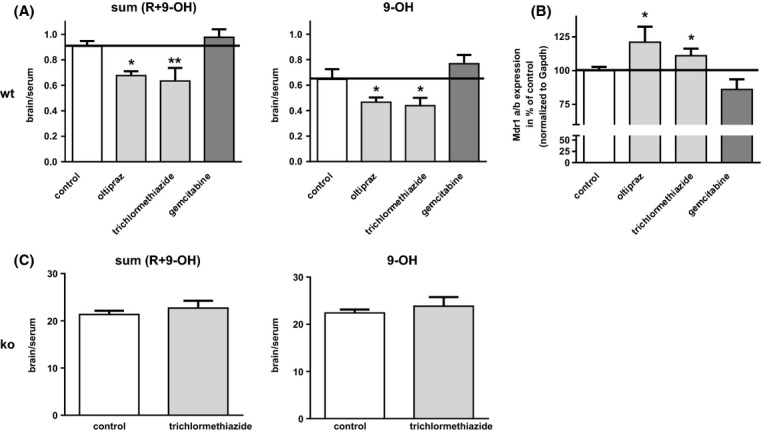
Effect of selected hits in wild-type and PGP knockout mice on risperidone transport into the brain. (A) Distribution of risperidone and its metabolite 9-OH-risperidone in candidate-drug pretreated mice. FVB/N male mice (wt) were treated with the respective drug 48 h by a daily injection (oltipraz: 30 mg/kg; trichlormethiazide: 8 mg/kg; gemcitabine: 20 mg/kg). DMSO-injected mice served as a control. Mice were subsequently injected i.p. with risperidone (3 mg/kg) and brains and serum collected after 3 h. Analysis of risperidone and the active metabolite was performed by HPLC and concentrations were determined by standard curves. Values obtained for brain samples were divided by those of serum and ratios were compared to those of control animals. Data represent mean ± standard error of samples from *n* ≥ 3 animals per group. (B) Expression of Mdr1a/b in total brain of mice pretreated with drug candidates. Mice were treated as described in A. Subsequently, mRNA was prepared from brain tissue homogenate and analyzed by real-time RT-PCR for Mdr1a and b mRNA. Values were calculated using a standard curve and normalized to Gapdh mRNA. Values represent mean ± standard error of samples from *n* ≥ 3 animals per group, performed in technical duplicates. (C) Impact of trichlormethiazide on risperidone/9-OH-risperidone transport in PGP knockout mice. Male PGP knockout (ko) mice were treated as described in A (*n* = 5 for control, *n* = 4 for ko). Samples from serum and brain homogenates were analyzed for risperidone and 9-OH-risperidone content by HPLC (unpaired student’s *t*-test versus control; all *P* > 0.05).

Results obtained from in vivo transport rates of risperidone in mice (Fig.5) are in accordance with cell culture transport experiments: oltipraz as well as trichlormethiazide reduced brain to serum levels of the metabolite 9-OH-risperidone or risperidone/9-OH-risperdidone as compared to solvent injected mice after a 3-h incubation period (Fig.5A). Gemcitabine only by trend increased the influx of risperidone or its metabolite as values did not reach significance. Analysis of total brain mRNA levels of both, Mdr1a and b, revealed that gemcitabine resulted in a 9% decrease as compared to DMSO-injected mice (Fig.5B) which is in accordance with the lack of influence on risperidone transport. Oltipraz treatment induced mRNA levels to about 130% of control animals and trichlormethiazide application to about 115%. To confirm that this rather small effect observed on gene expression of Mdr1a and b is responsible for modified risperidone transport in the mouse experiments, we repeated the approach in Mdr1a/b-ko-mice: brain to serum levels of those mice treated with trichlormethiazide regarding the sum of risperidone and its metabolite as well as the metabolite alone were indistinguishable from solvent-treated mice (Fig.5C). This indicates that expression of functional MDR1a/b is needed to mediate the influence of trichlormethiazide on transport across the blood–brain barrier.

## Discussion

In this article, we introduce a new dual screening assay for Mdr1a and 1b promoter activities. For human MDR1 gene, two distinct promoter regions have been described: the upstream (Chen et al. 2004) and a downstream promoter (Ueda et al. 1987a,b; Chen et al. 1990). Mouse genomic sequence reveals some similarity regarding the upstream promoter elements such as NF-IL6 (Raguz et al. 2008) but transcription seems to be dominantly controlled by the downstream promoter. Aberrant transcription from human upstream promoter occurs only in drug-selected cell lines and patients with, for example, relapsed lymphoma (Huff et al. 2005). Therefore, we decided to analyze the respective major downstream promoter regions Mdr1 a and b of murine origin. Using the HDAC inhibitor TSA and co-transfected expression plasmids for p53 and HSF-1, we were able to demonstrate that the dual assay results in comparable effects with even higher values to obtain as single performed measurements. For TSA an inducing potential on downstream promoter-driven Mdr1 mRNA synthesis has been reported for various cell lines using 1 *μ*mol/L of HDAC inhibitor (Balaguer et al. 2012) ranging from 500 to 8000% of control. This is in consistence with our observed induction of promoter activity in N2A cells even if we added TSA in a concentration of 15 nmol/L only. In silico analysis indicated potential HSF-1 binding sites in both promoter regions, while p53 binding position was identified in the Mdr1 b promoter only. Interestingly, overexpression of HSF-1 only induced Mdr1 b activity. HSF-1 and HSF-2 are able to act as heterodimers on regulatory DNA stretches. Because Mdr1 b in addition to HSF-1 binding site also comprises potential HSF-2 binding positions, we assume that this might be the reason for the observed selectivity. For human MDR1 promoter, an induction of expression via HSF-1 has been described (Vilaboa et al. 2000).

p53 is discussed controversially regarding impact on Mdr1 promoters: for example, a strong dependency on the cell line in which experiments are conducted has been found. In NIH-3T3, a lack of influence of wild-type p53 has been described (Chin et al. 1992); SW13 cells displayed a decrease and H358 as well as SW620 and 2780 cells reacted with a stimulated activity of a reporter (Goldsmith et al. 1995). This cell-type dependency of effect in addition to the absence of a p53 binding site at least in human MDR1 promoter region and the murine Mdr1a promoter sequence has led to the assumption of a rather indirect influence (e.g., Scotto et al. 1998). For example, a cooperative binding of p53-SP1 complex or interaction of p53 with Wt1 has been reported (overview in Millau et al. 2009). Therefore, the observation of p53 inducing both Mdr1a and b promoter in our assay seems plausible even if Mdr1a promoter sequence lacks a p53 binding motif. In sum, predictions from the in silico analysis of Mdr1 promoter sequences and the outcome of the reporter assay strongly suggest that an explorative analysis is needed for verifying regulatory influences.

Our dual assay represents a tool for time-resolved high-throughput-adaptable investigations. We analyzed 627 FDA-approved drugs for their impact on both promoters. About 90% of the tested substances remained without influence (93% for Mdr1a, 88% for Mdr1b). This is in accordance with other screening approaches in promoter analyses. Wright et al. (2010) report that only nine of 1040 substances influenced SOD1 promoter and for the HLA-B27 promoter only 5% of 12264 substances inherited modulating properties (Zhao et al. 2011). We identified 23 enhancers and four inhibitors that timely consistent regulated the two promoter activities, Mdr1a and b, while over 100 substances acted only on one of the regulatory sequences. As human MDR1 has been described to combine both gene properties (Silverton et al. 2011), it will be of interest to analyze the effect of the specific and generally acting drugs on its promoter. For gemcitabine, in vivo data only in tendency matched our data from reporter gene assay. This might be due to overlaying translational or epigenetic control mechanisms that have not been considered in our screening approach due to a lack of 3′UTR of the respective genes and sequences flanking the core promoters. For example, translational efficacy of Mdr1 has been demonstrated to be controlled by 5′ flanking regions in colon carcinoma cells (Gomez-Martinez et al. 2007). A potential discrepancy between drug-induced Mdr1 promoter activity and mRNA levels/transport activity has also been shown recently for TSA treatment of rat hepatoma cells (Sike et al. 2014). For trichlormethiazide as a newly identified Mdr1 inducer from the in vitro screening, we were able to demonstrate its effects on drug transport in primary endothelial barrier building cells. Effects obtained are similar to those of oltipraz, an already known inducer of gene expression. This transport impairing function was also confirmed in vivo by assessing risperidone levels in brain and periphery and dependency on expression of Mdr1 has been confirmed by our experiments in PGP ko mice. Trichlormethiazide is a diuretic drug, used for treatment of edema and hypertension. It inhibits sodium and chloride ion reabsorption from the distal tubules of the kidneys (Shimizu et al. 1988), increases the excretion of potassium (Takahashi et al. 2011) and thereby encourages water loss from the body. In contrast to trichlormethiazide-induced Mdr1 gene expression, it has been shown that dehydration or a high-salt diet in rats reduces expression of Mdr1 b in kidney (Morales et al. 2000). At least to our knowledge, a direct contribution to altered transcription factor levels by trichlormethiazide has not been reported. Therefore, an indirect regulation of Mdr1 gene expression driven by osmotic alterations might be suggested. Nevertheless, its property of increasing Mdr1 expression might interfere with therapeutic interventions.

## Conclusion

In summary, our dual promoter assay was able to reidentify oltipraz as a known Mdr1 transcriptional enhancer, and suggests trichlormethiazide as a new enhancer of Mdr1 expression. Analyzing comparative potential of drugs identified here to human promoter sequence response and also to upstream promoters will be of interest to further evaluate interference with blood–brain barrier function.

## Abbreviations

BBB: blood–brain barrier
FDA: Food and Drug Administration
HSF1/2: Heat-shock factor 1/2
Mdr1: Multidrug resistance protein 1 coding gene (murine)
MDR1: multidrug resistance protein 1 coding gene (human)
N2A: murine neuroblastoma cell line Neuro-2A
NF-IL6: nuclear factor for interleukin-6
PBEC: porcine brain endothelial cells
PGP: P-glycoprotein
RLU: relative light units
TSA: trichostatin A

## Supporting Information

**Figure S1.** Candidates for further investigations selected from screening with the dual Mdr1a/Mdr1b promoter assay were analyzed regarding EC/IC50. Values represent mean of promoter activities measured upon 48 h of incubation (two independent experiments; *n* ≥ 4), Values obtained for solvent-treated cells were set to 100%.

**Table S1.** Common regulators identified by dual Mdr1a/Mdr1b promoter assay. Values represent mean ± SD of promoter activities measured upon 24 or 48 h of incubation (*n* ≥ 3), normalized to values obtained for solvent-treated cells. Drugs further analyzed by in vitro and in vivo experiments are highlighted in gray. Final concentrations were as indicated.
