# Supplementary material for: Identification of trichlormethiazide as a Mdr1a/b gene expression enhancer via a dual secretion-based promoter assay
Source: Pharmacol Res Perspect. 2015 Jan 5;3(1):e00109. doi: 10.1002/prp2.109 (PMC4317239; doi:10.1002/prp2.109)

Supplementary Figure 1

Candidates for further investigations selected from screening with the dual Mdr1a/Mdr1b promoter assay were analyzed regarding EC/IC_{50}.
Values represent mean of promoter activities measured upon 48 hrs of incubation (two independent experiments; n≥4), Values obtained for solvent-treated cells were set to 100%.

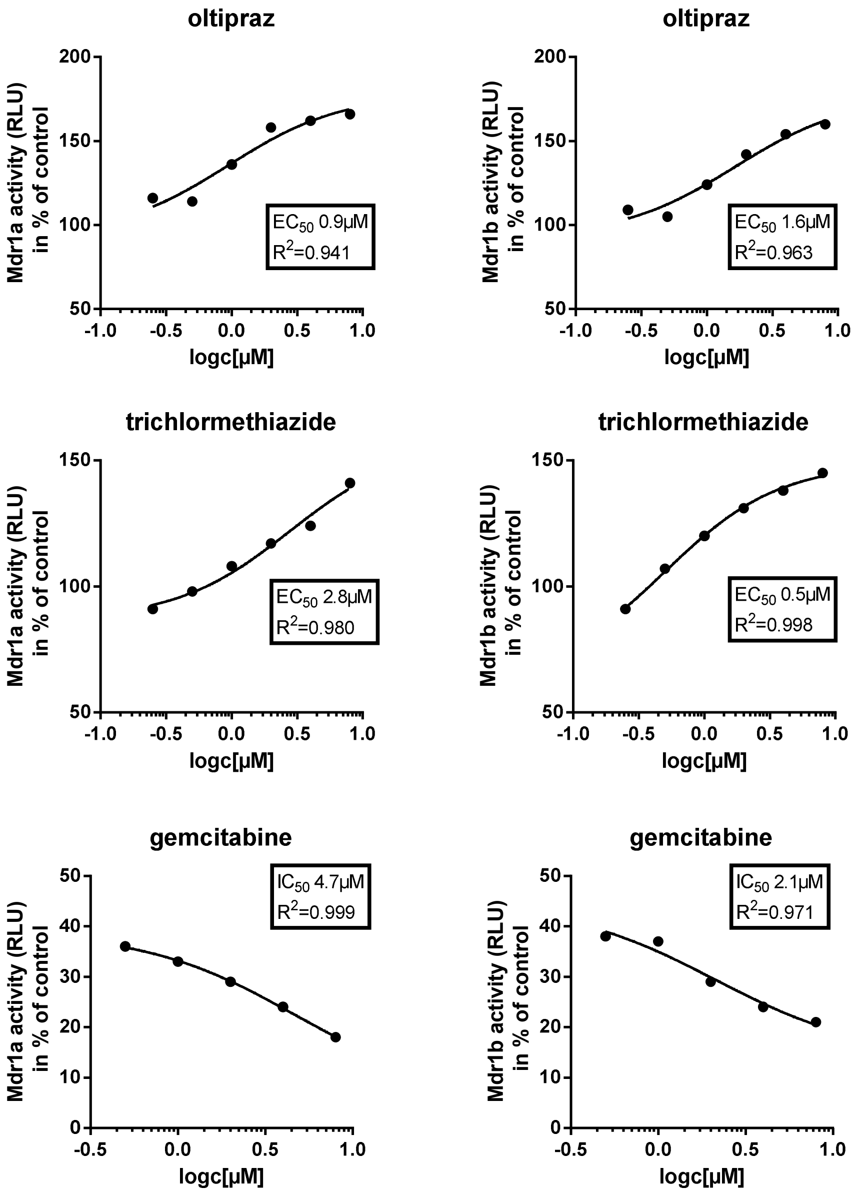

Supplement: Supplementary file 1 [file prp20003-e00109-sd1.pdf]
