# Supplementary material for: Identification of trichlormethiazide as a Mdr1a/b gene expression enhancer via a dual secretion-based promoter assay
Source: Pharmacol Res Perspect. 2015 Jan 5;3(1):e00109. doi: 10.1002/prp2.109 (PMC4317239; doi:10.1002/prp2.109)
Supplement: Supplementary file 2 [file prp20003-e00109-sd2.pdf]

Supplementary Table 1

		Mdr1a				Mdr1b			
		incubation period				incubation period			
**drug**	**[µM]**	24h		48h		24h		48h	
		MW	SD	MW	SD	MW	SD	MW	SD
acetylcholine Cl	1.46	147	34	142	8	185	12	222	24
alprenolol HCl	2.49	140	6	148	10	151	18	157	27
amoxapine	3.14	138	23	143	33	162	27	170	34
artesunate	3.84	59	15	30	6	47	9	25	6
butaclamol (+)	3.62	163	22	148	30	185	23	147	56
carbamylcholine Cl	1.47	135	4	142	17	137	10	144	20
cyproterone acetate	4.17	137	12	159	12	143	19	195	57
desloratadine	3.11	137	17	174	56	179	12	238	65
dextromethorphan HBr	2.71	132	7	148	43	198	5	224	61
esomeprazole potassium	3.45	149	41	168	33	163	29	182	28
estradiol	2.72	131	8	131	17	142	15	151	24
ethisterone	3.12	157	15	144	15	156	14	161	14
fenbufen	2.54	154	29	156	25	157	16	162	38
fenoldopam mesylate	3.06	155	30	195	28	161	36	189	14
gemcitabine HCl	2.63	42	7	43	15	37	7	40	15
idoxuridine	3.54	134	3	271	66	131	10	272	64
iloprost	3.60	174	43	145	35	179	49	171	30
imipenem	2.99	132	7	147	16	152	49	177	29
meglumine	1.95	132	3	142	12	144	12	148	46
mitoxantrone 2HCl	4.44	58	17	57	13	56	9	67	19
norepinephrine-(+)-tartrate l (-)	3.19	135	14	131	32	148	36	147	33
oltipraz	2.26	160	38	166	14	156	34	201	17
oxacillin sodium monohydrate	4.00	140	8	157	11	145	22	175	17
propafenone	3.42	130	15	144	12	158	18	149	21
rapamycin	9.14	34	6	45	25	40	24	29	8
sulpiride s (-)	3.41	135	11	134	13	159	19	153	13
trichlormethiazide	3.81	149	31	176	21	160	23	195	31

Common regulators identified by dual Mdr1a/Mdr1b promoter assay. Values represent mean ± SD of promoter activities measured upon 24 or 48 hrs of incubation ($n \geq 3$), normalized to values obtained for solvent-treated cells. Drugs further analyzed by *in vitro* and *in v*ivo experiments are highlighted in grey. Final concentrations were as indicated.
